# Role of Male Sex Partners in HIV Risk of Adolescent Girls and Young Women in Mozambique

**DOI:** 10.9745/GHSP-D-19-00117

**Published:** 2019-09-23

**Authors:** Jenifer Chapman, Nena do Nascimento, Mahua Mandal

**Affiliations:** a MEASURE Evaluation, Palladium, Mozambique.; b MEASURE Evaluation, Palladium, Washington, DC, USA.; c MEASURE Evaluation, University of North Carolina, Chapel Hill, NC, USA.

## Abstract

Adolescent girls and young women (AGYW) ages 15–24 years are disproportionately affected by HIV/AIDS, particularly in East and Southern Africa. One strategy to reduce HIV among AGYW, proposed through the Determined, Resilient, Empowered, AIDS-free, Mentored, and Safe (DREAMS) Initiative, is to prevent and manage HIV among their male sexual partners. To implement this strategy and reach men, programs need information about AGYW's potential sexual partners at the local level. To support DREAMS programming in Mozambique, we undertook a study to characterize this population of men in 3 districts with ongoing DREAMS programming. In mid-2017 we conducted 15 focus group discussions with AGYW (N=102) and a venue-based intercept survey of men (N=1,140). Male sexual partners of AGYW who took the survey were diverse in age, education level, and socioeconomic status. Older AGYW focus group participants sought partners who could provide for them financially. Multiple sexual partnerships and inconsistent condom use were widely reported, with AGYW emphasizing that gender norms disempowered them from negotiating condom use. Reported condom use varied by AGYW and male-partner demographic characteristics, as well as by their relationship type. Condom use rates were much higher than national and regional estimates. AGYW who were less educated/not-in-school, were pregnant, or single mothers were particularly disempowered in sexual relationships. Less educated men were less likely to use condoms than educated men, and condom use was least likely in marriage. Study findings underscore the importance of reaching the diversity of male sexual partners of AGYW with HIV services as part of a strategy to reduce HIV risk among AGYW. They also support an enhanced focus on female-controlled HIV prevention methods that do not require negotiation with a male partner and special efforts to reach out-of-school/less educated AGYW, as well as pregnant AGYW and single mothers.

## Abstract (precis)

Efforts to prevent HIV among adolescent girls and young women (AGYW) should focus on providing male sexual partners of AGYW with HIV prevention, testing, and treatment programming and providing AGYW, particularly those who are less educated, pregnant, or single mothers, with prevention methods that do not require negotiating safer sex with their partners.

## INTRODUCTION

Adolescent girls and young women (AGYW) ages 15–24 years are disproportionately affected by HIV/AIDS.^1^^,^^2^ In sub-Saharan African countries with generalized HIV epidemics, adolescence marks an increase in HIV prevalence and the emergence of gender disparities in HIV.^3^ Recent estimates from 7 African countries found that the prevalence of HIV among girls and women ages 15–25 is more than twice that of their male counterparts.^4^

Despite the epidemiological and human rights imperative to help AGYW remain AIDS-free, programming in this area has resulted in uneven and slow progress in reducing HIV infection rates. Fewer than half of AGYW living with HIV know their status,^4^ and treatment uptake and global viral suppression rates among adolescents and young people, especially among females, are extremely low.^4^^–^^7^ Furthermore, although other age groups have experienced declines in AIDS-related deaths, adolescent AIDS-related deaths increased by about 50% globally between 2005–2012.^3^

Over recent years, vulnerable AGYW have participated in direct prevention interventions, such as small-group educational and skills-building initiatives, that have been shown to strengthen confidence, self-efficacy, access to sexual reproductive health services, and other related outcomes^8^^–^^11^. Despite these efforts, AGYW remain at high HIV risk. Another strategy to reduce HIV among AGYW, proposed through the Determined, Resilient, Empowered, AIDS-free, Mentored, and Safe (DREAMS) Initiative and the new MenStar Coalition (both funded by consortia of global partners), is to prevent HIV among currently HIV-negative male sexual partners of AGYW and reduce the transmission by male partners who are HIV-positive (by controlling their viral load). This strategy requires understanding the profile of sexual partners of AGYW, particularly males at high risk of acquiring or transmitting HIV. Although research on cross-generational and transactional sex among AGYW and older male partners has been ongoing in sub-Saharan Africa,^2^^,^^12^^–^^16^ understanding the characteristics of the male sexual partners of AGYW in general is a new area of inquiry.^17^

Reducing HIV among adolescent girls and young women requires understanding the profile of their potential partners.

In Mozambique, which has benefitted from DREAMS funding and targeted prevention efforts, HIV prevalence among AGYW is 9.8%—more than 3 times higher than among males of the same age group (3.2%).^18^ AIDSInfo/SPECTRUM estimates that 28,000 AGYW were newly infected with HIV in 2017, comprising one-quarter of new infections among those ages 15 years and older. To support DREAMS work in-country, we undertook a study to characterize men who have recently engaged in sexual activity with AGYW and identify relationship characteristics that influence sexual risk-taking behavior.

## METHODS

This study was conducted in 3 districts in Mozambique—Quelimane, Beira, and Xai-Xai—that receive targeted AGYW prevention support services. These urban/peri-urban districts are located in the northern, central, and southern regions of the country, respectively, with high rates of HIV prevalence. Quelimane and Beira are both port cities that sit along major inland transport routes. Xai Xai district is a major point along the major North/South transport corridor.

We conducted a mixed-methods study involving focus groups with AGYW and a venue-based survey of males who had AGYW as sexual partners. Ethical clearance was obtained from Health Media Labs, Inc., in the United States, and the Comitê Nacional de Bioética para a Sáude in Mozambique.

### Data Collection and Analysis

#### Focus Group Discussions

In each study district, we conducted 5 focus group discussions (FGDs) in mid-2017 with the following subgroups of AGYW: (1) in school, ages 15–17 years; (2) out of school, ages 18–19, and not pregnant, postpartum, or breastfeeding; (3) out of school, ages 15–19, married, and pregnant, postpartum, or breastfeeding; (4) ages 20–24, and not pregnant, postpartum, or breastfeeding; and (5) ages 20–24, and pregnant, postpartum, or breastfeeding. The subgroups were defined based on target subpopulations for the DREAMS intervention districts.

We developed a topic guide to elicit information from AGYW on the characteristics of male sexual partners of AGYW, how risk-taking varies by type of relationship and sexual partner, and gender dynamics in sexual relationships. With the support of local DREAMS implementing partners, trained female data collectors recruited AGYW participants from girls' groups, schools, and health centers. Before collecting information, data collectors sought and documented informed consent/assent from each participant (and from the caregivers of minors).

In Beira, FGDs were held at a local partner facility; in Quelimane, at a secondary school; and in Xai Xai, at a primary school. In Xai-Xai and Beira, discussions were held in a mix of Portuguese and the local languages (Changana and Sena, respectively). In Quelimane, the discussion was held in Chuwabe and Portuguese, and a local resident translated for the participants and study team. All discussions were audio-recorded.

Data collectors transcribed the FGD recordings, translated all local languages to Portuguese, and expanded on the transcripts with field notes. All identifying data were redacted to protect participants' privacy. Two qualitative analysts followed a collaborative qualitative analysis approach through the 5 interrelated steps for data analysis: reading, coding, displaying, reducing, and interpreting.^19^^,^^20^

#### Venue-Based Survey

In mid-2017, we conducted a short quantitative survey in public venues with men ages 18 and older to identify and characterize males who have AGYW as sexual partners, spending one week in each district.

We surveyed men ages 18 and older to identify and characterize males who have adolescent girls and young women as partners.

To ensure we were powered to calculate our key indicators, we used prevalence of male circumcision—a key DREAMS intervention—to calculate the sample size. Using a conservative estimate of 51%, with a 5% margin of error, an estimated design effect of 2.0, and an estimated response rate of 80%, we calculated a required study sample size of 930 men who had recently engaged in sex with an AGYW (we use this abbreviation in the singular to refer to any member of this demographic group). We aimed to recruit men from at least 10 venues in each of the 3 study locations.

To select venues, we elicited information during FGDs with young women on where we might find different types of AGYW sexual partners (e.g., husbands, boyfriends, casual partners, transactional partners). We created a list of venues, including bars, schools, markets, and beaches, in each district. Then, we reviewed this list with stakeholder reference groups (comprising members of the government and local implementing partners), which we formed in each district to advise on study implementation and to improve data use. Nearby venues were combined, closed venues were removed from the list, and several venues were added. We worked with establishment owners/staff and local government to gather permissions to recruit at the venues selected. During this process, we determined the best time of day to collect data from each venue and developed the field schedule.

At the venues, data collectors approached men to participate in a 15-minute interviewer-administered, tablet-based survey. We applied a “take all” approach at every venue, meaning that we approached all men in the venue to participate. Men who were 18 years or older and who appeared sober were eligible to participate.

Once eligibility and willingness to participate were determined, the data collector and potential participant moved to a private location near the venue (generally, just outside it) to document informed consent and, if granted, administer the survey. The survey instrument had 2 parts. Part 1 focused on respondent demographics with a final question about whether the respondent had recently had sex with an AGYW. Respondents who reported having had sex with an AGYW in the past 12 months proceeded to part 2, which included questions about sexual partnerships, demographics (theirs and their partners'), HIV testing and knowledge of HIV status, male circumcision, condom use, participation in HIV services, and preferences for and barriers to HIV services. Data collection continued until at least 310 men in each study site had completed part 2 of the questionnaire.

We analyzed the data in Stata 15 in several steps. First, we used frequency distributions to explore characteristics of the full sample of men who reported ever having sex. Next, we compared characteristics of men who reported having had sex with an AGYW in the past 12 months with those who had not. All subsequent analyses were restricted to men who reported having had sex with an AGYW in the past 12 months. A series of bivariate analyses were conducted to examine associations between characteristics and risk-taking behaviors of AGYW and their male partners. For each research question, we also conducted multivariable logistic regression to examine characteristics of male partners and AGYW that were associated with men's use of condoms.

## RESULTS

A total of 102 AGYW participated in 15 FGDs across 3 study locations. Demographic characteristics aligned to recruitment criteria across the 5 subgroups of AGYW (see methods). Of the 1,520 men who were approached, 1,176 men (77.4%) consented to participate in the survey and were eligible. We recruited men from 38 venues (11 in Beira, 13 in Xai-Xai, and 14 in Quelimane).

### How AGYW Characterize Their Male Sexual Partners

Participants across all focus groups described sexual relationships with male partners diverse in age, educational attainment, marital status, and employment status.

Participants said it was “normal” to have a partner one's own age, but also reported that AGYW of all ages had sexual relationships with older men. Generally, females ages 15–19 years said that girls their age dated men who were slightly older (in their early 20s or 30s). Young women ages 20–24 years described dating slightly older men (in their late 20s and early 30s), and some in both age groups described dating significantly older men (in their 40s, 50s, or 60s).

Participants in all groups expressed a strong preference for men who could offer material and economic security and who would take responsibility for pregnancy (with these concepts often being linked). Respondents typically associated these traits with older men. One respondent described a friend who was dating an older Mozambican businessman working in South Africa:

*He brings back 8,000, 9,000 [US $130–150]—I've seen the money… he is married … and I would say he is around 40 years old and she is 16.* —AGYW, 15–17 years, in-school, Beira

Adolescents and young women preferred partners who offered material and economic security.

An adolescent said that she would rather be with an older man than someone her own age because of the monetary support an older man could provide her:

*I am with my boyfriend and I find an older man who can give me everything—he buys me hair pieces, clothes, you do not even know how much. So, I am with that man, because he is able to satisfy all of my wants, because I am with him for his money … He will always be buying me expensive phones. So, when you see that [that this man can provide], and the fact that your boyfriend cannot give you any money at all, pretty soon you are running to the older man.* —AGYW, 18–19 years, out-of-school, Quelimane

AGYW across all subgroups reported that their sexual partners had different levels of education, ranging from none to primary, secondary, and university education. The only exception was in FGDs with young women ages 20–24 years who were pregnant, postpartum, and/or breastfeeding. Across the 3 regions, these AGYW noted that their sexual partners either had no education, minimal education, or no more than secondary education.

Respondents explained that AGYW had sexual relationships both with married and single men. Younger and single AGYW participants noted that many AGYW their age may have preferred to date married men because these relationships provided them with material goods without leading to a long-term commitment. Participants noted that single women ages 20–24 years were more often looking for a serious relationship that could lead to marriage; therefore, they were less interested in married men.

FGD participants reported that AGYW had sexual partners who do both skilled and low-skilled work, but AGYW indicated they valued male sexual partners with high-skilled employment. An adolescent explained the high value she and her peers placed on men who work for the State. She recalled hearing a girl her age who bragged about her boyfriend's employment:

*I know a girl [who dates a professor], and so when she is out with friends she says, ‘I go out with someone who works for the State.’ I don't know if she is really mostly interested in dating someone who works for the State, I don't know if that man says that he wants nothing to do with her when he goes out with friends. That would make her more humble, since she says she dates someone who works for the State. I really want nothing to do with her.* —AGYW, 18–19 years, out-of-school, Xai-Xai

Younger FGD participants, both those in and out of school, said that sexual relationships between girls and schoolteachers were very common. These relationships were described as coercive because teachers typically promised good grades or other benefits in exchange for sex. One adolescent said:

*Other girls here, even in this school, go out with teachers. They even say, “If you want to pass you have to go out with him.” So, why spend the whole school year working hard to get good grades, if he will fail you at the end of the year?… This school is full of teachers who hook up with their students.* —AGYW, 15–17 years, in-school, Quelimane

### How Men With AGYW as Sexual Partners Characterize Themselves

Eighty-six percent (n=981) of men surveyed reported recently (within the past 12 months) having had sex with an AGYW. Men who reported recent sex with AGYW were, on average, 27 years old (range 18–58 years). One-quarter (23.9%) of these men reported at least some primary education, one-quarter had some secondary education, one-quarter had completed secondary education, and one-quarter had at least some tertiary education. One-third (31.7%) were studying at the time of the survey. Eighty-four percent of men reporting an AGYW sexual partner had worked in the 7 days prior to the survey (90.5% during the 12 months prior). Respondents reported various occupations and a range of monthly incomes. Approximately half (50.9%) were married/cohabiting and a further 38.1% reported having a steady girlfriend (of any age) – 89% of all men reporting recent sex with an AGYW reported a steady partner (Table 1).

**TABLE 1.** Characteristics of Men Reporting Sex With AGYW in Mozambique, by AGYW Partner Status

	Has AGYW Partner (n=981)	Does Not Have AGYW Partner (n=159)
Age, years, mean (standard error)***	27.33 (.22)	41.5 (.76)
Age, years, range	(18, 58)	(26, 64)
Currently studying,*** No. (%)	311 (31.70)	17 (12.23)
**Highest level of completed education, No. (%)**		
Less than primary***	60 (6.12)	18 (12.95)
Completed primary	174 (17.74)	24 (17.27)
Some secondary	251 (25.59)	29 (20.86)
Completed secondary*	256 (26.10)	24 (17.27)
Completed more than secondary	240 (24.46)	44 (31.65)
**Employment, No. (%)**		
Worked in the past 7 days†	824 (84.00)	131 (94.24)
Worked in the past 12 months**	888 (90.52)	137 (99.56)
**Income, No. (%)**		
<$1,000	41 (4.18)	4 (2.88)
$1,000–4,999**	295 (30.07)	21 (15.11)
$5,000–9,999	257 (26.20)	36 (25.90)
$10,000–19,999	170 (17.33)	31 (22.30)
$20,000-39,999*	78 (7.95)	22 (15.83)
≥$40,000***	28 (2.85)	17 (12.23)
Missing data	112 (11.42)	8 (5.76)
**Marital status, No. (%)**		
Single***	470 (47.91)	10 (7.19)
Married or living together***	499 (50.87)	114 (82.01)
Widowed/divorced***	12 (1.22)	15 (10.79)

Abbreviation: AGYW, adolescent girls and young women.

† *P*<.10;

* *P*<.05;

** *P*<.01;

*** *P*<.001.

Compared to men who did not report recent sex with an AGYW, those who did were younger (on average: 27 years versus 43 years, *P*<.001), less likely to be married (50.9% versus 82.0%, *P*<.001), and less likely to be less educated (6.1% versus 13.0%, *P*<.001); more likely to have worked in the 12 months before the survey (90.5% versus 99.6%, *P*<.01); and more likely to be currently studying (31.7% versus 12.2%, *P*<.001). In a multivariable analysis, only age, having a steady partner (married or cohabitating), and education were significantly associated with recent sex with an AGYW. After controlling for all other demographics (except profession), younger men (adjusted odds ratio [AOR]: 0.84, *P*<.001) were more likely to report a recent AGYW sexual partner, and men with low educational attainment (less than primary) were less likely to report a recent AGYW sexual partner (AOR: 0.37, *P*<.05) (Table 1).

Men reporting recent sex with an AGYW reported extremely high rates of HIV testing compared to national and regional rates—82.8% reported ever being tested for HIV. About three-quarters of the sample (76.0%) reported being circumcised.

### Sexual Risk Behavior

We examined numbers of sexual partners and condom use, as reported by men and AGYW.

#### Multiple Sexual Partners

Half (50.1%) of the men who had AGYW partners reported having had 3 or more sex partners in the previous 12 months (45.5% of those who were married). Married FGD participants noted that their husbands often had relationships with other women. One young woman described her husband's extramarital relationships:

*My husband has lots of women. Each woman has her own home, and they are boyfriend and girlfriend.* —AGYW, 20–24 years, pregnant, postpartum and/or breastfeeding, Quelimane

Half of males surveyed reported having 3 or more partners in the previous 12 months.

Although a few of the married AGYW described monogamous relationships, more married participants reported that they and their married AGYW friends had a boyfriend as well as a husband. One participant said,

*This happens a lot. I have a friend who has a husband, but she also has 3 boyfriends.* —AGYW, 18–19 years, out-of-school, Beira

Focus group participants said that married women were motivated to have relationships with other men in part for economic reasons, particularly if their husbands were not providing for them. One adolescent said:

*They [other women] have to find a boyfriend to give them money.* —AGYW, 18–19 years, out-of-school, Quelimane

Focus group participants also noted that extramarital relations were more common when husbands work far away from the home.

#### Inconsistent Condom Use

Sixty percent (60.3%) of men surveyed reported condom use at last sex, and 41% reported consistent (i.e., always) condom use in the last 12 months. Condom use was related to male partner characteristics, AGYW characteristics, the type of relationship, and gender norms.

Of the males surveyed, 41% reported consistent condom use in the last 12 months.

**Male partner characteristics.** Men who reported condom use at last sex, compared to those who did not, were more likely to be single (55.1% versus 37%, *P*<.001), currently studying (38% versus 22.1%, *P*<.001), and have tertiary education (29.1% versus 17.5%, *P*<.001), and less likely to be working (81.4% versus 87.9%, *P*<.01) (Table 2). After controlling for all demographics except profession, men currently studying had higher odds of reporting condom use at last sex (AOR: 1.64, *P*<.01) and consistent condom use (AOR: 1.68, *P*<.001). Men with some primary education or no education had lower odds of reporting condom use at last sex (AOR: 0.61, *P*<.05) and consistent condom use (AOR: 0.53, *P*<.01), and, compared to single men, married men had lower odds of reporting condom use at last sex (AOR: 0.36, *P*<.05) and consistent condom use (AOR: 0.24, *P*<.001).

**TABLE 2.** Male Partner Characteristics Associated With Men's Reported Condom Use, Mozambique

	Condom Use at Last Sex			Condom Use Frequency in Last 12 Months		
	Yes (n=592)	No (n=389)	*P* Value	Always (n=389)	Inconsistent (n=558)	*P* Value
Age, years, mean (standard error)	27.16 (.29)	27.60 (.34)		26.79 (.36)	27.72 (.29)	
Age, years, range	(18, 58)	(18, 52)		(18, 58)	(18, 52)	
Currently studying, %	38.01	22.11	***	40.87	25.27	***
**Education, %**						
Less than primary	4.73	8.23	*	3.60	8.24	**
Completed primary	13.68	23.91	***	13.62	21.15	**
Some secondary	25.34	25.96		26.48	24.91	
Completed secondary	27.20	24.42		27.51	24.91	
Completed more than secondary	29.05	17.48	***	28.79	20.79	*
**Marital status, %**						
Single	55.07	37.02	***	58.35	40.14	***
Married or living together	43.07	62.72	***	40.62	58.78	***
Widowed/divorced	1.86	0.26	*	1.03	1.08	

* *P*<.05;

** *P*<.01;

*** *P*<.001; *P* values are based on chi-square statistics from bivariate analysis.

**AGYW characteristics.** Men reporting that their most recent AGYW sexual partner was younger than 20 years old, compared to those who reported AGYW sexual partners ages 20–24 years, were more likely to report condom use at last sex (64.8% versus 58.1%, *P*<.05) and consistent condom use (51.9% versus 35.6%, *P*<.001). Similarly, men reporting that their most recent AGYW sexual partner was employed, compared to those who reported an unemployed AGYW sexual partner, were more likely to report condom use at last sex (66.8% versus 58.2%, *P*<.05). However, we did not detect a difference in consistent condom use between these groups. Men reporting that their most recent AGYW sexual partner was a mother and/or pregnant were less likely to report condom use at last sex, compared to those reporting sex with AGYW who were not mothers or pregnant at the time (45.8% versus 69.7%, *P*<.001), and less likely to report consistent condom use (24.7% versus 52.1%, *P*<.001). Men reporting that their most recent AGYW sexual partner was school-age but not attending school or attending primary school were less likely to report condom use at last sex and consistent condom use compared to men reporting that their most recent AGYW sexual partner was attending secondary or tertiary school (Table 3).

**TABLE 3.** Men's Risk-Taking Behaviors, by Men's Reported AGYW Sex Partner Demographic Characteristics, Mozambique

	AGYW Demographic Characteristics												
	Current Age			Employment			Pregnant/Mother			Education			
	13–19 years (n=332)	20–25 years (n=646)	*P* Value	Employed (n=226)	Not employed (n=750)	*P* Value	Pregnant/mother (n=395)	Not pregnant/not mother (n=575)	*P* Value	Attending primary (n=104)	Attending secondary (n=524)	Attending higher education (n =107)	School age but not attending school (n=82)
Condom use at last sex													
Yes (n=591)	64.8	58.1	*P* <.05	66.8	58.2	*P* <.05	45.8	69.7	*P* <.001	47.1**	66.8***	69.2*	54.2*
No (n=388)	35.2	41.9		33.2	41.8		54.2	30.3		52.9	33.2	30.8	45.8
Condom use frequency in last 12 months													
Consistent (n=389)	51.9	35.6	*P* <.001	41.4	41.1		24.7	52.1	*P* <.001	29.8*	47.8***	43.7	26.2**
Inconsistent (n=557)	48.2	64.3		58.6	58.9		75.3	47.9		70.2	52.1	56.3	61.0

Abbreviation: AGYW, adolescent girls and young women.

† *P*<.10;

* *P*<.05;

** *P*<.01;

*** *P*<.001; *P* values are based on chi-square statistics from bivariate analysis.

In some focus groups, women said that a single, pregnant woman left without any support would sometimes engage in riskier sexual behavior (e.g., not using condoms, sleeping with many men) in an effort to find a partner who could take care of her. An AGYW said:

*They [pregnant single women] go after truck drivers, those people with money, they go after them to get what their child needs, if the father doesn't take on his role to take care of the child.* —AGYW, 15–19 years, married and pregnant/postpartum/breastfeeding, Quelimane

Another girl said:

*You have a child with him, but he denies it [that he is the father]. You don't have a way to sustain your child. You'll have to go find a man to get you some money, so you can buy Omo [detergent] for your child. Because children pee every day, and you have to always be cleaning [their clothes], and you need Omo to wash them.* —AGYW, in-school, 15-17 years, Quelimane

**Type of relationship.** In Table 4, we present data on men's reported risk-taking behavior by the type of relationship they had with their most recent AGYW sexual partner. Men were more likely to report condom use at last sex with AGYW sexual partners that they just met (79.5%) and AGYW to whom they reported giving money for sex (67.5%) compared to their wives (24.2%, *P*<.01). Men whose AGYW partners were not their wives also had significantly higher odds of consistent condom use; for example, men whose AGYW partners were steady (but not live-in) partners had 5 times the odds of always using a condom (AOR: 5.13, *P*<.001). Men whose AGYW partners were sex workers had 15 times the odds (AOR:15.59, *P*<.05) of always using a condom compared to men who were married or living with their AGYW partners.

**TABLE 4.** Men's Risk-Taking Behaviors, by Men's Reported Type of Relationship With AGYW Sex Partner, Mozambique

	Relationship Type							
	Wife/live-in partner (n=190)	Steady partner (n=379)	Irregular partner (n=120)	Ex-partner (n=17)	Friend (n=209)	Colleague/student (n=12)	Just met (n=44)	Sex worker (n=7)
**Condom use at last sex**								
Yes (n=591)	24.2***	64.9*	72.5*	64.7	71.9***	83.3	79.5**	85.7
No (n=388)	75.8	35.1	27.5	35.3	28.1	16.7	20.5	14.3
**Condom use frequency in last 12 months**								
Consistent (n=389)	8.4***	43.4	55.2**	41.2	54.6***	58.3	64.7**	75.0
Inconsistent (n=557)	91.6	56.7	44.8	58.9	45.4	41.7	35.3	25

Abbreviation: AGYW, adolescent girls and young women.

† *P*<.10;

* *P*<.05;

** *P*<.01;

*** *P*<.001; *P* values are based on chi-square statistics from bivariate analysis.

AGYW described differences in condom-use behavior by relationship type, but explained that the decision to use a condom was almost always driven by the male partner. AGYW said that they never use condoms with their husbands, but that they sometimes (albeit infrequently) used condoms with their boyfriends or casual partners. Married respondents said if a woman asked her husband to use a condom, he would suspect her of infidelity or of having HIV or another sexually transmitted infection. A young woman said:

*For me to use it [a condom], it has to be him who broaches the subject; the boss has to say, “Let's use it.” I know that he will get suspicious [if I suggest using a condom] … “Why today are you talking about us using this?” … “She must be sick.”* —AGYW< 20–24 years, Beira

Another young woman said of her husband:

*He'll leave me at home suffering and will go find another woman [if I insist on using a condom]… I will offend him, because he's in charge.* —AGYW, 20–24 years, Quelimane

A few younger girls in each region said that condom use was more common with boyfriends their age and that they themselves use condoms. One adolescent said:

*[Condoms also] prevent illness, unwanted pregnancy…. For us, this age, it is normal [to use them].* —AGYW, 18–19 years, out-of-school, Beira

Younger participants in Xai-Xai said that younger men (often their serious boyfriends) were much more open to the idea of using condoms, and much better informed. One AGYW said:

*… at school there are always lectures [on condoms] …. So, they [men their age] pay attention.* —AGYW, 18–19 years, out-of-school, Xai-Xai

**Gender norms.** Most AGYW expressed feeling powerless in persuading boyfriends and casual partners to use condoms, fearing the partners might leave them. One adolescent said:

*I arrive in the home of my boyfriend and he doesn't have a condom, and I also don't have one. So, I don't know if it is fear or embarrassment … but always when my boyfriend says that there isn't a condom, the girl always shuts up and has sex without a condom. … It's rare that my boyfriend actually says, “No, no, let's do it without a condom.” He will always threaten me: “If you love me, we have to have sex without a condom.” We always give in … we have to prove we love him … we shut up. We have this fear, like, I will lose him, I have to accept to do it how he wants it.* —AGYW, 18–19 years, out-of-school, Xai-Xai

Most adolescents and young women expressed feeling powerless in persuading partners to use condoms, fearing the partners might leave them.

Participants described coercion around not using condoms and specifically how older boyfriends with means offer gifts to encourage AGYW not to use condoms. One adolescent said:

*[Men say] I'll give you a phone, I'll give you everything that you want today [if we can have sex without a condom].*—AGYW, 18–19 years, out-of-school, Quelimane

Similarly, another adolescent said:

*Us girls … we have sex without prevention, we forget about condoms, we only have sex in the way the man wants it, a man who is the age of our fathers, or even with boys our age … [and] they could be infected with HIV. But we want the material goods he has, we don't want love, and we hurry to date him. Afterwards, we have sex without a condom, we don't use prevention methods. And then we get HIV without realizing it, and girls die without knowing what's killing them.* —AGYW, 15–17 years, in-school, Xai-Xai

## DISCUSSION

This study is the first to document the characteristics of male sexual partners of AGYW in Mozambique and factors that relate to HIV risk-taking behavior. The mixed-methods approach employed in this study allows for 2 perspectives, providing complementary qualitative data from AGYW and quantitative data from men with AGYW as sexual partners.

Both qualitative and quantitative data described a diversity of male sexual partners in terms of educational attainment, employment status, income, and marital status. Whereas quantitative data from surveyed men suggested that male partners were more likely to be young and employed, qualitative data from women's FGDs indicated that AGYW had male partners who were their age and older, as well as employed and unemployed partners. Qualitative data also suggested that while AGYW had male sexual partners across all categories of demographic characteristics, women strongly preferred men who could offer material and economic security, as well as take responsibility for pregnancy.

Qualitative data from women revealed that male school teachers were sexual partners of school-age AGYW, a finding that is consistent with studies in other sub-Saharan African contexts.^21^^–^^23^ This finding underscores the importance of providing male teachers, especially in secondary schools, with HIV prevention messages and interventions and putting in place a series of safe-guarding mechanisms, awareness-raising campaigns, and reporting and punishment protocols and policies. Teachers must be appropriately trained and screened, and widespread information campaigns could be deployed to expose this issue nationally. We encourage the use of anonymous or protected reporting mechanisms, such as *Linha Fala Criança*―a helpline already in place in Mozambique for children experiencing abuse. Enforcement mechanisms could be created through school and government authorities to punish teachers for having sex with students, especially minors. We recommend that the Ministries of Health, Education, and Justice work closely together to develop a plan to address this issue at national and subnational levels. This is particularly crucial because of the substantial evidence that staying in school is a protective factor for HIV among AGYW.^24^^–^^27^

Male partners reported higher than expected rates of circumcision and uptake of HIV testing services. According to national data, the proportion of men ages 15–49 who reported being circumcised varied between 20.1% and 47.6% in study provinces, and the proportion of men ages 15–49 who reported an HIV test in the past 12 months and who had received the results of that test varied between 15.9% and 31.8% in study provinces.^18^ This could be owing to our sampling strategy that focused on urban areas where services were near and readily available (perhaps mobile testing was offered at the recruitment venues we used), the effect of concentrated DREAMS programming in the study sites or social desirability bias: that is, men may have answered that they had tested, because they thought that was what interviewers wanted to hear.

Overall, we found that sexual relationships between AGYW and their male partners were characterized by high risk for HIV. Many AGYW and male sexual partners of AGYW reported multiple sexual relationships in the past 3 months, regardless of marital status, in line with general population data.^18^ In the current study, social norms, economic needs, and distance between marital couples were all cited as reasons contributing to the uptake of multiple partners. Another ethnographic study of young women in Maputo found that, in the context of high unemployment and limited economic opportunities for women, engaging in multiple and transactional sexual relationships created a pathway for women to gain financial and material resources.^16^

We found that relationships between adolescent girls and young women and their male partners were characterized by high risk for HIV.

Further, most men reported using condoms inconsistently, and men with less education had significantly lower odds of reporting consistent condom use. This was similarly reported in a study of miners in Mozambique.^28^ Men's reported condom use also differed based on the demographic characteristics of AGYW. Men were less likely to use condoms with AGYW who were out of school or had low educational attainment. Out-of-school and primary-attending AGYW were likely to have less self-efficacy, and thus less power in sexual relationships, than their in-school and secondary-school attending counterparts. A study conducted by Patrão and McIntyre (2017) in Mozambique reported similar findings on the relationship between the education level of AGYW and their condom-use self-efficacy.^29^

Surprisingly, condom use was more commonly reported in sexual partnerships with AGYW who were younger (ages 15–19 years) rather than older (ages 20–24 years). AGYW in our study noted that condom use was more common with boyfriends similar in age than with older—often married—boyfriends. This finding echoes a recent study in Mozambique on contraceptive use^30^ and may explain why condom use was more frequently reported by men with younger rather than older AGYW sexual partners. Finally, condom use at last sex was less likely to be reported in sexual relationships with mothers/pregnant women than it was in relationships with AGYW who were not pregnant/mothers. One explanation may be that AGYW were pregnant or mothers were married to their male partners, and condom use was low within marriage. Another explanation may be that condoms were used primarily to prevent pregnancy, not HIV, in these relationships, and once AGYW became pregnant or gave birth, they no longer used condoms. One 7-country study of HIV transmission among sero-discordant couples found that condoms were less likely to be used with pregnant than nonpregnant women, and this was the case whether the woman or the man in the couple was HIV-positive.^31^ This may be because pregnancy prevention was a higher priority than HIV prevention for men and women.^32^ The finding underscores the need to develop interventions promoting condom use with pregnant women and girls to reduce HIV transmission.

Condom use was particularly low in the context of marriage. Married AGYW said that condom use (and even the decision to have sex) was almost always determined by their male partners, and that their ability to negotiate safer sex was extremely limited. This narrative is not a new one in southern Africa^33^^–^^36^ or in Mozambique.^37^

AGYW described a context in which gender norms and power dynamics create barriers in their ability to negotiate condom use with sexual partners. Economic vulnerability further undermined AGYW's ability to protect themselves. In our study, AGYW reported less willingness to negotiate condom use in relationships in which the male partner provided money or other benefits to them or their families. Pregnant AGYW and mothers who lacked a partner who financially provided for them and their baby may have been particularly at risk of engaging in HIV risk behavior because of increased financial needs. Capurchande and colleagues also found that parenthood amplified economic and social pressures that AGYW faced and may increase their risk-taking behaviors.^30^

Adolescent girls and young women reported less willingness to negotiate condom use in relationships in which the male partner provided money or other benefits.

### Limitations

This study has limited generalizability; samples of AGYW and male sexual partners were drawn from 3 urban/peri-urban areas with current DREAMS programming. Study districts have experienced, on average, a 45% decline in HIV prevalence among AGYW between 2015 and 2017.^38^ Furthermore, the quantitative portion of the study was not a representative sample of all male sexual partners of AGYW in study districts, owing to recruitment methods. Data were subject to self-reporting and recall biases. We did not collect biomarkers to validate the demographic and behavioral data. Survey respondents may have over-reported condom use, and gender norms may have influenced males to over-report whether they had sex with an AGYW in the past year. Also, men were asked only to report on their most recent sexual partner, limiting analyses.

## CONCLUSIONS

The sexual partners of AGYW in Mozambique have diverse demographic profiles, from young students to older, married men and high schoolteachers. Although this study did not assess HIV prevalence, sexual partnerships may be characterized as high-risk—condom use was infrequently reported and inconsistent, and many men described concurrent sexual partnerships. Findings confirmed strong gender norms favoring the male as the decision maker in sexual relationships, as well as social norms that impeded condom use in marriage. AGYW who were less educated or who were pregnant/single mothers may have been at increased risk of practicing unsafe sexual behaviors, owing to lower negotiating power and social pressure. However, there are reasons for optimism. Condom use was more commonly reported by men with educated AGYW partners, and AGYW reported using condoms with their school-going boyfriends more routinely than with older partners. This indicated that school-based HIV and pregnancy prevention campaigns may be having a positive impact—a subject that requires further study. Nevertheless, the economic incentives to participate in unsafe sexual practices persist. Results confirmed the importance of reaching the male sexual partners of AGYW with HIV prevention, testing, and treatment programming and providing AGYW with prevention methods, such as pre-exposure prophylaxis, that do not require negotiating safer sex with their partners.

Condom use was infrequently reported and inconsistent, and many men described concurrent partnerships.

## Notes

**Peer Reviewed**

**Funding:** Financial support was provided by the United States Agency for International Development (USAID) under the terms of MEASURE Evaluation cooperative agreement AID-OAA-L-14-00004. This work was funded by USAID and the United States President's Emergency Plan for AIDS Relief from the DREAMS Initiative to MEASURE Evaluation based at the University of North Carolina at Chapel Hill. Views expressed are not necessarily those of USAID or the United States government.

**Disclaimer:** This paper was prepared by the authors with funding from USAID. The information provided in this report is not official U.S. Government information and does not necessarily reflect the views or positions of the USAID or the U.S. Government.
